# On the Fragmentation of Ni(II) β-Diketonate-Diamine Complexes as Molecular Precursors for NiO Films: A Theoretical and Experimental Investigation

**DOI:** 10.3390/molecules29030642

**Published:** 2024-01-30

**Authors:** Cristiano Invernizzi, Gloria Tabacchi, Roberta Seraglia, Mattia Benedet, Marco Roverso, Chiara Maccato, Sara Bogialli, Davide Barreca, Ettore Fois

**Affiliations:** 1 Department of Science and High Technology, Insubria University and INSTM, 22100 Como, Italy; cinvernizzi@uninsubria.it (C.I.); ettore.fois@uninsubria.it (E.F.); 2 CNR-ICMATE and INSTM, Department of Chemical Sciences, Padova University, 35131 Padova, Italy; roberta.seraglia@cnr.it (R.S.); mattia.benedet@phd.unipd.it (M.B.); marco.roverso@unipd.it (M.R.); chiara.maccato@unipd.it (C.M.); sara.bogialli@unipd.it (S.B.); 3 Department of Chemical Sciences, Padova University and INSTM, 35131 Padova, Italy

**Keywords:** transition metal complexes, oxide nanomaterials, NiO, molecular precursors, chemical vapor deposition, density functional theory, simulations

## Abstract

NiO-based nanomaterials have attracted considerable interest for different applications, which have stimulated the implementation of various synthetic approaches aimed at modulating their chemico-physical properties. In this regard, their bottom-up preparation starting from suitable precursors plays an important role, although a molecular-level insight into their reactivity remains an open issue to be properly tackled. In the present study, we focused on the fragmentation of Ni(II) diketonate-diamine adducts, of interest as vapor-phase precursors for Ni(II) oxide systems, by combining electrospray ionization mass spectrometry (ESI-MS) with multiple collisional experiments (ESI-MS^n^) and theoretical calculations. The outcomes of this investigation revealed common features in the fragmentation pattern of the target compounds: (i) in the first fragmentation, the three complexes yield analogous base-peak cations by losing a negatively charged diketonate moiety; in these cations, Ni-O and Ni-N interactions are stronger and the Ni positive charge is lower than in the parent neutral complexes; (ii) the tendency of ligand electronic charge to migrate towards Ni further increases in the subsequent fragmentation, leading to the formation of a tetracoordinated Ni environment featuring an interesting cation-π intramolecular interaction.

## 1. Introduction

Nanomaterials based on NiO, a multi-functional *p*-type semiconductor with a wide band gap (3.5 eV) [1], have been the object of numerous studies in view of their various technological end-uses, among which are electrochromic devices [2,3,4,5,6,7], solar cells [8,9,10,11,12], gas sensors [13,14,15,16,17], and heterogeneous catalysts for a variety of processes [18,19,20,21,22,23,24,25,26]. This wide perspective of attractive utilization has triggered interest in their preparation by different strategies [1], among which sol-gel and chemical vapor deposition (CVD) [27,28,29], that are endowed with several degrees of freedom to tailor material structure, chemical composition, and morphology. In particular, the CVD family encompasses a variety of routes based on heterogeneous nucleation and growth processes, significantly influenced by the characteristics of the starting molecular compounds [30,31,32].

In this context, metal β-diketonate complexes are among the most widely used precursors for the CVD of metal oxide films and nanomaterials [33,34]. They present significant flexibility for the modification of their chemico-physical properties by varying the steric hindrance, features, and dimensions of the ligand backbone [33]. Nevertheless, as regards the CVD of NiO, the conventional [Ni(acac)_2_] [22,35] and [Ni(thd)_2_] [36,37] (Hacac = 2,4-pentanedione; Hthd = 2,2,6,6-tetramethyl-3,5-heptanedionate) suffer from concurrent problems, including their high melting points and limited gas phase stability [33,35,38,39,40], that hinder the achievement of a precise process control and prevent their straightforward large-scale use.

In order to circumvent these problems, following our previous works on homologous first-row transition metal complexes [30,31,41,42,43,44,45], we have focused on the preparation and characterization of Ni(II) β-diketonate-diamine compounds of the general formula NiL_2_TMEDA [HL = 1,1,1,-trifluoro-2,4-pentanedionate (tfa), 2,2-dimethyl-6,6,7,7,8,8,8-heptafluoro-3,5-octanedionate (fod), or thd; TMEDA = *N*,*N*,*N*′,*N*′-tetramethylethylenediamine]. The results of a comprehensive experimental and theoretical investigation [46,47] highlighted that these compounds offer several amenable characteristics as CVD precursors of NiO films with modular properties. Insights into the compound reactivity were previously obtained by electron ionization mass spectrometry (EI-MS) [46], typically considered more appropriate for the investigation of CVD precursor gas-phase reactivity, especially in plasma-assisted processes [46]. Nonetheless, the hard ionization conditions lead to the destruction of specific ions, which is diagnostic of compound fragmentation [31]. Conversely, such information can be usefully obtained by soft ionization methods like electrospray ionization mass spectrometry (ESI-MS) [41,42,43], enabling a deeper knowledge on precursor decomposition behavior.

**Scheme 1:**
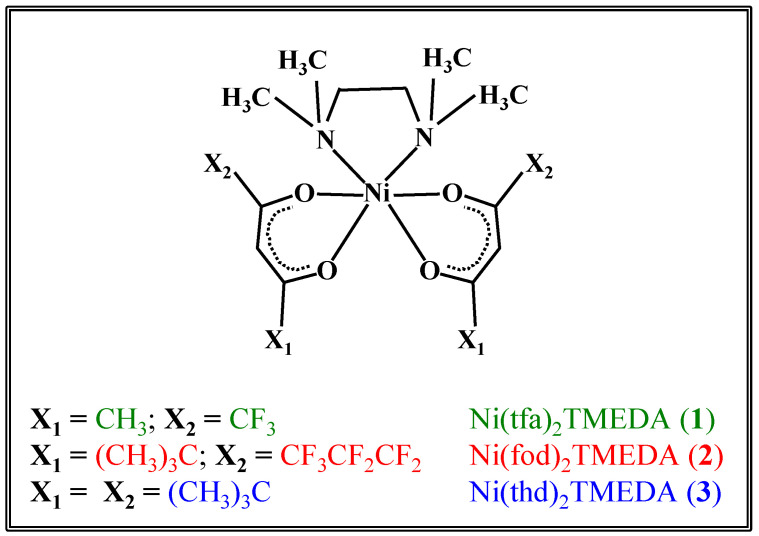
Sketch of the structures of the three Ni(II) diketonate-diamine adducts targeted in the present work.

In the present study, the fragmentation behavior of the three compounds Ni(tfa)_2_TMEDA (**1**), Ni(fod)_2_TMEDA (**2**), and Ni(thd)_2_TMEDA (**3**) (Scheme 1) is investigated by electrospray ionization high resolution mass spectrometry (ESI-HRMS), ESI-MS^n^ experiments, and theoretical investigations. A detailed computational study based on density functional theory (DFT) calculations was carried out with the aim of thoroughly elucidating the molecular and electronic structure of NiL_2_TMEDA-derived fragments (where L = tfa, fod, or thd). The outcomes reveal how metal–ligand bonding schemes evolve upon passing from the pristine precursor to the fragments identified by MS analyses. The obtained data provide for the first time a valuable picture of the gas-phase fragmentation mechanisms of such compounds as a function of the ligand nature, as well as information on both the geometric and electronic structures of the fragmentation products, setting the basis for a molecular-level understanding of their chemical reactivity.

## 2. Results

### 2.1. ESI-MS Experiments

**Figure 1:**
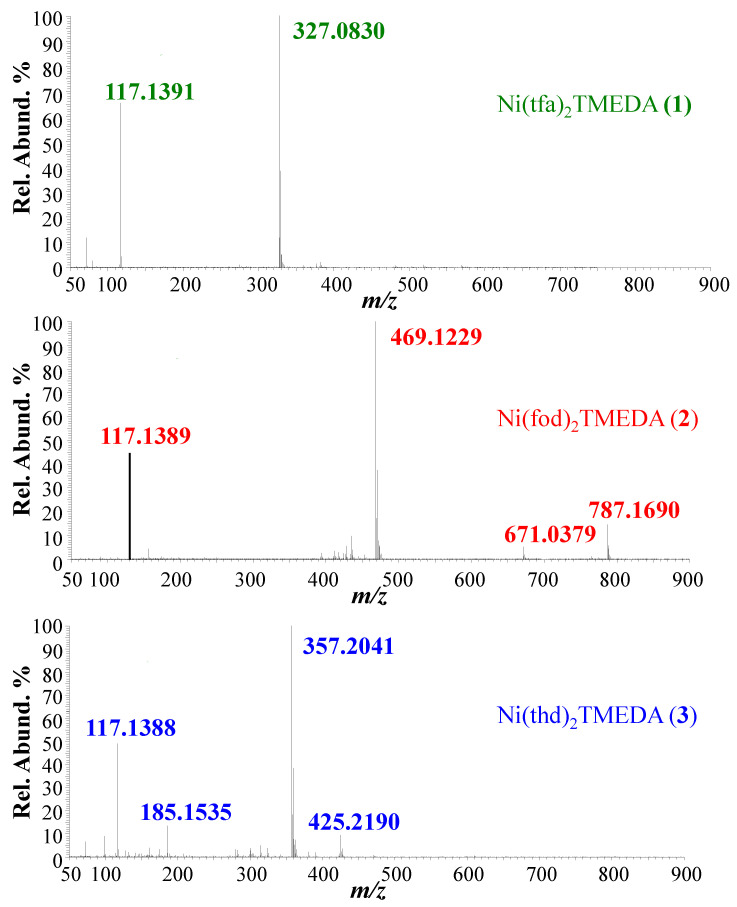
Positive ESI-HRMS spectra of methanolic solution of compounds **1**–**3**.

**Figure 2:**
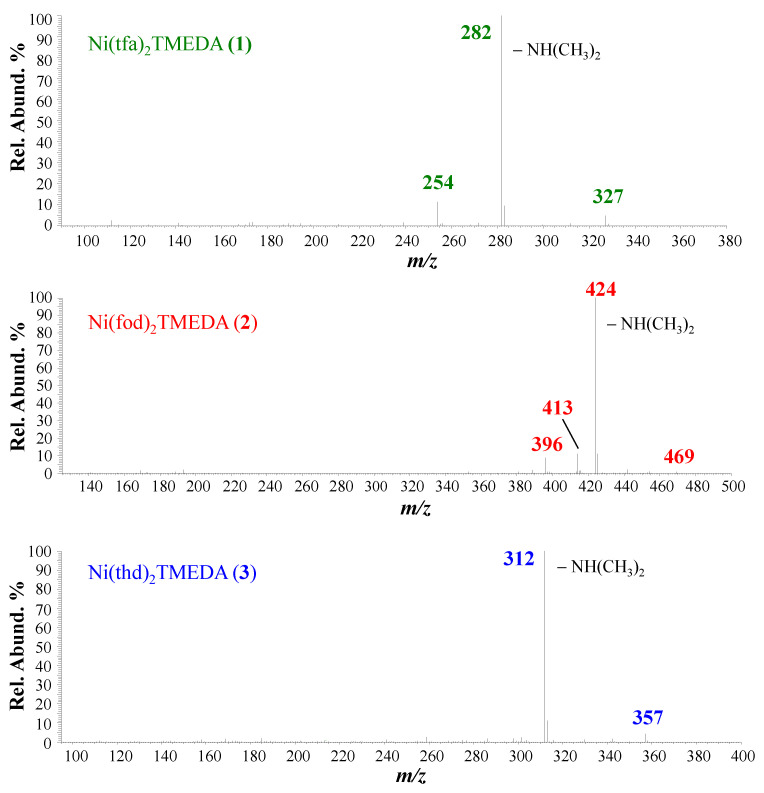
ESI-MS^2^ spectra of [M−L]^+^ ions for compounds **1**, **2**, and **3**.

Positive ion mode ESI-HRMS mass spectra for the three complexes are displayed in Figure 1, whereas the identification of the main fragments, *m*/*z* ratios, and relative abundances are provided in Table 1. In all cases, the base peak (*m*/*z* = 327.0830, 469.1229, and 357.2041 for **1**, **2,** and **3**, respectively) was related to [M−L]^+^ species (where M indicates compound **1**, **2**, or **3**) arising from the loss of a β-diketonate ligand L. Ions due to the protonated diamine [TMEDA+H]^+^ were detected at *m*/*z* = 117.1390, with a relative abundance > 45% for all three compounds. Only for **2**, the molecular ion was detected as a sodium adduct at *m*/*z* = 787.1690 ([M+Na]^+^), together with [M−TMEDA+Na]^+^ species formed through TMEDA loss (*m*/*z* = 671.0379). The presence of [M+Na]^+^ for **2** can be attributed to a good efficiency in the formation of Na^+^ adducts under ESI-MS conditions, promoted by the partial negative charge of CF_2_CF_2_CF_3_ groups due to fluorine atoms [48]. For compound **3**, the signal at *m*/*z* = 425.2190 was attributed to TMEDA loss from the protonated molecular ion (not detectable). MS/MS experiments (see Figure 2) on [M−L]^+^ ions revealed further decomposition due to TMEDA-related rearrangements. In particular, NH(CH_3_)_2_ loss from the TMEDA moiety turned out to be the most favored fragmentation process, resulting in the formation of ions at *m*/*z* = 282, 424, and 312 for **1**, **2,** and **3**, respectively. Furthermore, for fluorinated compounds **1** and **2**, the loss of -CH_3_CH_2_N(CH_3_)_2_ from [M−L]^+^ led to ionic species at *m*/*z* = 254 and 396, respectively. Only for [M−L]^+^ of compound **2**, an additional rearrangement of the fod ligand led to species at *m*/*z* = 413, due to C(CH_3_)_2_=CH_2_ loss.

### 2.2. DFT Calculations

**Figure 3:**
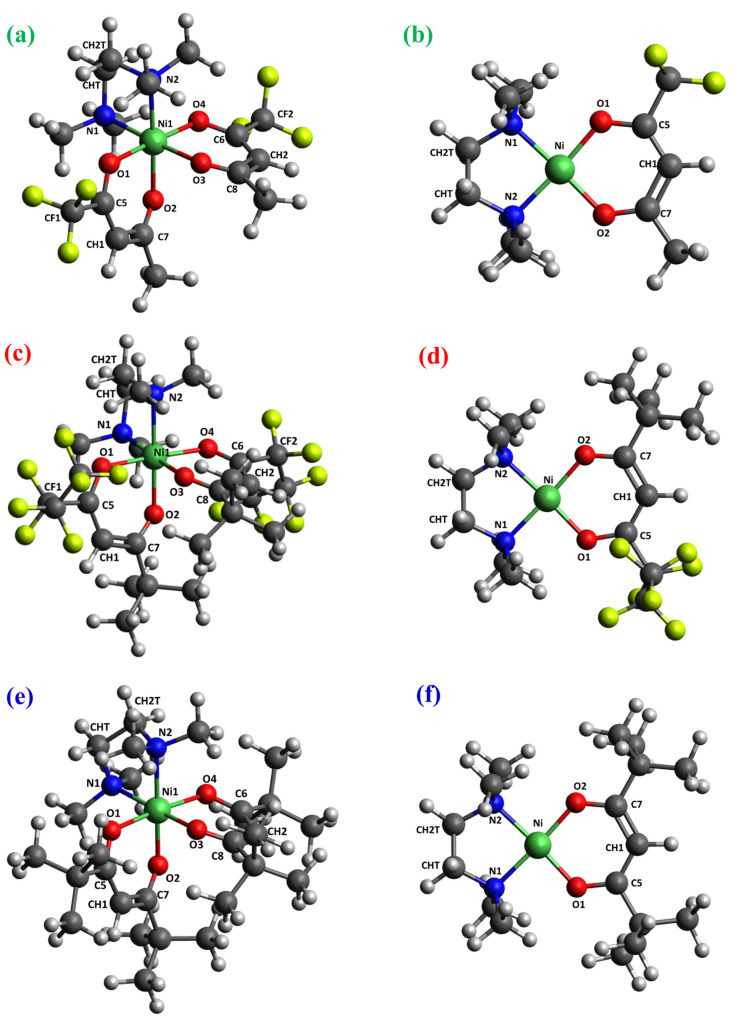
DFT-computed minimum energy structures for [Ni(L)_2_TMEDA] with L = tfa (**a**), fod (**c**), thd (**e**), and for [Ni(L)TMEDA]^+^ with L = tfa (**b**), fod (**d**), and thd (**f**). Color codes: green = Ni; yellow = F; blue = N; red = O; grey = C; white = H.

To elucidate the molecular structures of fragment ions detected in MS experiments, DFT calculations were performed, starting with the computation of the minimum energy structures of the three neutral complexes [Ni(L)_2_TMEDA] (Figure 3). Additionally, all fragments discussed in the previous section were subjected to structural optimization. Since, in electrospray ionization/mass spectrometry, the ions are ejected from the charged droplets of solvent through many desolvation steps in the ion source region, and consequently, only gas-phase ions arrive at the analyzer [49,50], all of the detected ions observed and described in this manuscript should be considered non-solvated. Accordingly, no solvation effects were considered in the DFT calculations. Concerning the choice of DFT approximation, the M06 hybrid meta-functional by Zhao and Truhlar was selected [51], in consideration of its broad applicability and good accuracy “across-the-board” for transition metal compounds [52]. In particular, this functional provided a good description of several complexes of the [M(L)_2_TMEDA] family (M = Fe, Co, Cu, Zn) [53], including the detailed fragmentation behavior of [Cu(hfa)_2_TMEDA] in ESI-MS experiments [45].

**Figure 4:**
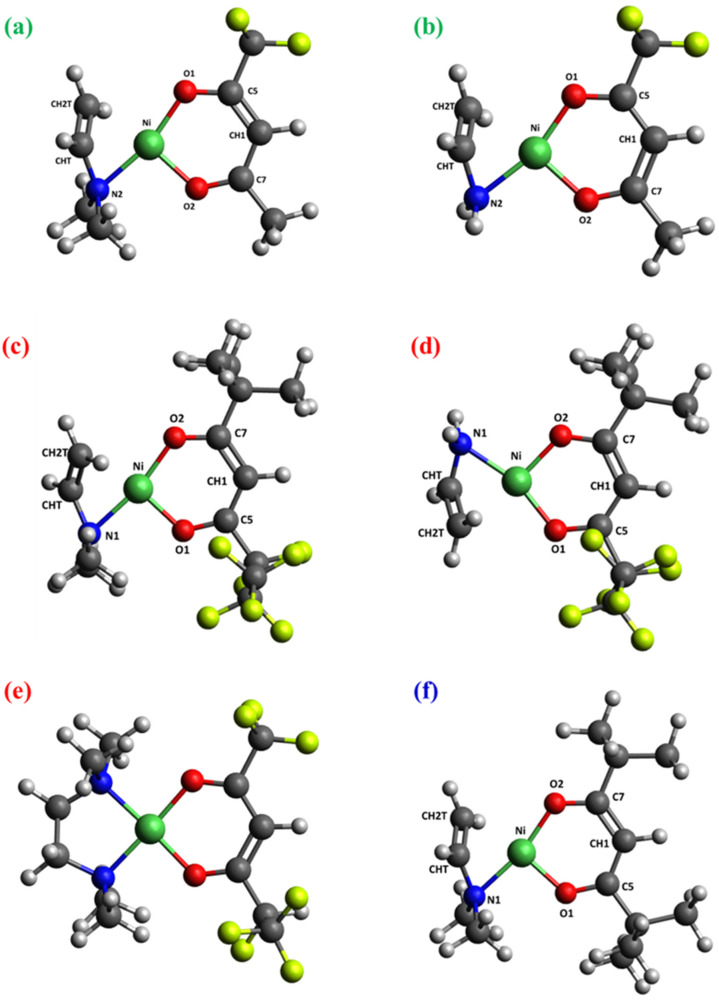
Optimized structures of: (**a**) [Ni(tfa)TMEDA]^+^-NH(CH_3_)_2_; (**b**) [Ni(tfa)TMEDA]^+^-CH_3_CH_2_N(CH_3_)_2_; (**c**) [Ni(fod)TMEDA]^+^-NH(CH_3_)_2_; (**d**) [Ni(fod)TMEDA]^+^-CH_3_CH_2_N(CH_3_)_2_; (**e**) [Ni(fod)TMEDA]^+^-(CH_3_)_2_C=CH_2_, due to an additional fod ligand rearrangement; (**f**) [Ni(thd)TMEDA]^+^-NH(CH_3_)_2_. Color codes as in Figure 3.

The optimized geometries of neutral complexes and fragment ions are displayed in Figure 3, Figure 4, Figures S1 and S2, while relevant geometrical parameters and electronic properties are reported in Table 2, Table 3 and Table 4 and Table S1. A graphical representation of the optimized structures for the parent neutral complexes is proposed in Figure 3a,c,e. Since all ESI-HRMS spectra showed a base peak due to the loss of a β-diketonate ligand L, a search for the minimum energy structures of [M−L]^+^ species was performed, starting from neutral complexes and removing a β-diketonate ligand. The resulting [Ni(L)TMEDA]^+^ optimized geometries are displayed in Figure 3b,d,f.

The molecular geometries of the three neutral complexes are similar, and the structural parameters are close to those computed using another density functional approximation [46]. In all cases, Ni exhibits a slightly distorted octahedral coordination environment, characterized by Ni-O distances shorter than the Ni-N ones (Table 2), suggesting nickel–oxygen interactions stronger than nickel–nitrogen ones. This hypothesis is substantiated by the bond order (BO) trend, since Ni-O BOs are systematically higher than Ni-N ones for all three complexes (Table 2). Interestingly, whereas BO values for Ni-O bonds are quite similar, the Ni-N bond order for the non-fluorinated compound **3**, bearing two tert-butyl groups per diketonate ligand, is slightly lower than for fluorinated complexes **1** and **2**. These data indicate that, whereas F-containing groups promote an increase in Ni-N bond strength, the presence of tert-butyl substituents has the opposite effect. Nevertheless, other than these slight differences, both molecular geometry and electronic structure underlying the metal–ligand bonding scheme show a strong similarity among the three compounds (see Figure 3 and Table 2). This result might rationalize the similar fragmentation behavior of the target complexes, in particular the presence of [M−L]^+^ as the base peak in all ESI-HRMS spectra (Figure 1).

Similar observations hold for the minimum energy structures calculated for [M−L]^+^ fragments, characterized by very similar geometric arrangements and metal–ligand bonding schemes. Specifically, all three ions are characterized by a square-planar geometry (Figure 3b,d,f) typical of tetra-coordinated Ni(II) complexes in the singlet spin state. In addition, both geometrical parameters and BO values show only very slight differences upon passing from **1** to **2** to **3** (Table 2). This result is supported by natural bond orbital (NBO) analysis performed on the electronic structure of the three [M−L]^+^ fragments. In particular, the data in Table S2 indicate a very strong similarity of the NBOs localized on metal–ligand bonds. Irrespective of the nature of the diketonate ligand, all NBOs have σ-character. While the bonding components [BD(σ)] are mostly localized on the ligand’s N and O atoms (≈90%), the antibonding components [BD(σ*)] are predominantly localized on Ni. Taken together, NBO data indicate that electron charge is transferred from ligands to Ni. This finding is further clarified by the representation of bonding NBOs (Figures S3–S5), highlighting the net σ-character as well as Ni’s participation in the bonding scheme. Such a close similarity in the electronic structure suggests that all three ions possess an analogous stability and may all easily form in ESI-HRMS conditions.

Notably, for all three complexes, Ni-O and Ni-N distances undergo a significant shortening upon passing from neutral compounds to [M−L]^+^ fragments. This variation is accompanied by the considerable increase in the corresponding bond orders, which are nearly doubled in the fragment ions. Hence, in terms of the metal–ligand bonding, the loss of a diketonate ligand results in significantly strengthened Ni-TMEDA interactions and Ni-O bonds of the remaining diketonate moiety.

A useful feature of NBO analysis is the opportunity for partitioning the electronic density between specific portions of a given molecule, thus enabling the estimation of the total charge on each. In this case, it is particularly instructive to inspect the total NBO charges localized on Ni, diketonate ligand L, and TMEDA (Table 3). Interestingly, the data show that the positive charge on Ni decreases by ≈25–30% on passing from neutral complexes to [M−L]^+^ cations. This result, which may appear counterintuitive due to the fragments’ positive charge, may be explained by considering that electronic charge donation from L and TMEDA to Ni significantly increases upon the loss of a diketonate ligand. Indeed, the total negative charge on L decreases by 50%, whereas the total (positive) charge on TMEDA is more than doubled (Table 3). Hence, these data indicate that all [M−L]^+^ species are stabilized by a strong electronic density donation from ligands towards Ni. This effect is slightly more pronounced in the fragment derived from compound **3**, due to the higher electron-donor character of the two tert-butyl substituents on the diketonate moiety with respect to fluorine-containing groups, present in compounds **1** and **2**.

In addition to the main peak common to the three complexes, ESI-HRMS spectra indicated the formation of other cations (see Figure 1 and Table 1), for which a careful computational search of minimum energy structures was also performed. Graphical representations of optimized geometries of [M+Na]^+^, [M−TMEDA+Na]^+^, [M−TMEDA+H]^+^, [HL+H]^+^, and [TMEDA+H]^+^ are shown in Figure S1, whereas selected geometrical parameters are reported in Table S1. It should be pointed out that Na^+^ is present only in the ESI-HRMS spectra of the fluorine-richest compound **2**. In [M+Na]^+^, Ni maintains its octahedral environment in spite of Na^+^ entrance in the second coordination shell (Figure S1a). Specifically, three O atoms are located at coordination distances from sodium (average Na-O distance = 2.416 Å), which is also interacting with a fluorine atom (Na-F distance = 2.441 Å). The consequent octahedral Ni environment distortion is evidenced by the lengthening of Ni-O and Ni-N bonds with respect to the neutral Ni(fod)_2_TMEDA. This effect is particularly relevant for the bonds involving the three oxygen atoms also coordinated with Na^+^ (Table S1). Similarly, the ion [M−TMEDA+Na]^+^, featuring a distorted square-planar Ni coordination, is characterized by significant O-Na^+^ and F-Na^+^ interactions (Table S1, Figure S1b). In a different way, [M−TMEDA+H]^+^, namely, [Ni(thd)_2_+H]^+^, is protonated at the CH1 carbon. For this ion, two structures very close in energy (ΔE = 0.32 kcal/mol) have been found (Figure S1c,d). The most stable one exhibits a distorted tetrahedral arrangement around the Ni center and triplet spin multiplicity. On the other hand, the least stable structure is characterized by a square-planar Ni coordination geometry and a singlet spin multiplicity. Due to the modest energy difference, both structures might be formed during the ESI process. The presence of the protonated diketonate species [HL+H]^+^ was detected only in the case of the more electron-donor thd ligand, whose optimized structure is characterized by the protonation of both carbonylic groups (Figure S1e). On the other hand, the protonated ligand [TMEDA+H]^+^ is detected in the fragmentation of all complexes, and in this case, the protonation occurs on one of the nitrogen atoms (see Figure S1f).

As far as MS^2^ spectra are concerned, attention was focused on the subsequent fragmentation of [M−L]^+^ cations. Even in this case, the spectra of the three cations presented common features, in particular the formation of [M−L]^+^—NH(CH_3_)_2_ species. The theoretical search for minimum energy structures for these moieties led to the results depicted in Figure 4a,c,f. Remarkably, these cations have very similar geometries and Ni–ligand bonding patterns. Whereas the diketonate remains coordinated to Ni with both its O atoms, only one N atom (the one that survived the fragmentation) is linked to the metal center. Nevertheless, Ni maintains its nearly square-planar coordination geometry thanks to the interaction with a double C=C bond formed upon -NH(CH_3_)_2_ release. Specifically, the Ni-CHT and Ni-CH2T distances become ≈2 Å in these fragments (see Table 4). In addition, the data reported in Table 4 indicate a net shortening of the CHT-CH2T bond length (Figure 4) in comparison to the same distance in [M−L]^+^ species (Table 2). This phenomenon is accompanied by a significant increase in the CHT-CH2T bond order (from ≈1 to >1.5; see Table 2 and Table 4). Correspondingly, Ni-CHT and Ni-CH2T BO values become comparable to those of Ni-O/Ni-N bonds, indicating appreciable Ni-C interactions that are greater for the terminal C atom (CH2T), which is closer to Ni. These observations are common to the three cations, underscoring once again the similarity of metal–ligand bonding schemes for systems derived from compounds **1**, **2**, **3**.

The loss of a -CH_3_CH_2_N(CH_3_)_2_ group was observed only for fluorinated [M−L]^+^ species derived from **1** and **2**. In these cations (Figure 4b,d), the bonding pattern around Ni is practically the same as in the [M−L]^+^-NH(CH_3_)_2_ species previously discussed. The square planar tetra-coordinated Ni structure is characterized by two Ni-O bonds, one Ni-N bond, and a cation-π intramolecular interaction between the Ni center and the CHT=CH2T double bond. Figures S6–S10 display the relevant orbitals involved in this interaction, whereas Tables S3 and S4 report the quantitative NBO analysis. As can be observed, the Ni cation actively participates in this interaction, with the π structure predominantly localized on the CHT=CH2T double bond. 

As regards compound **2**, species corresponding to the loss of a CH_3_C=CH_2_ group from [Ni(fod)TMEDA]^+^ ([Ni(fod)TMEDA]^+^-(CH_3_)_2_C=CH_2_) were observed in the ESI-MS spectra. Their stoichiometry indicates a rearrangement of the fod ligand (see Figure 1 and Table 1). To identify the structure of these cations, geometry optimizations were carried out on different hypothetical structures possessing such a stoichiometry. Figure S2 reports the six most stable structures, along with their relative stability (including the zero-point contribution). The most stable structure (Figure S2a) presents a distribution of F atoms on both diketonate ligand sides. A similar feature is also shown by other isomers characterized by modest energy differences with respect to the minimum energy structure.

Finally, the trend of the Bader charges on passing from the neutral complex to the positive ions further evidences common features in the fragmentation of the three complexes (see Tables S5–S8). The obtained data highlight an electron displacement from ligands to the Ni center, whose positive charge is significantly depleted during the fragmentation, in line with the findings obtained from NBO analysis.

## 3. Materials and Methods

### 3.1. Experimental Details 

Ni(L)_2_TMEDA complexes (L = tfa, fod, thd) were synthesized following recently reported procedures, and their characterization by elemental analyses, IR, and various complementary techniques has already been reported [46]. Electrospray ionization high-resolution mass spectrometric (ESI-HRMS) data were acquired by a Q Exactive™ hybrid quadrupole-Orbitrap™ mass spectrometer (ThermoFisher Scientific, Waltham, MA, USA) in positive ion mode (resolution = 70,000; sheath gas N_2_ at 10 psi; spray voltage = 3.5 kV; and capillary temperature 280 °C). Orbitrap MS calibration was performed using a standard ThermoFisher Scientific Pierce^®^ ESI positive-ion calibration solution. Low-resolution electrospray ionization mass spectrometry (ESI-MS) analyses were performed using a LCQFleet ion trap instrument (ThermoFisher Scientific), operating in positive ion mode. The spray voltage and capillary temperature were 4.0 kV and 250 °C, respectively. In both cases, 10^−6^ M solutions of the target compounds were introduced by direct infusion using a syringe pump (flow rate = 10 μL × min^−1^). MS^n^ experiments were performed by applying a supplementary radio frequency (RF) voltage to the end caps of the ion trap (5 V peak-to-peak).

### 3.2. Computational Details

Density functional theory calculations were performed on [Ni(L)_2_TMEDA] (with L = tfa, fod, thd) and their fragments. Specifically, the M06 functional [51] was adopted, and the Gaussian 09 code was employed [54]. This functional was chosen for its capability of delivering a good description of the molecular and electronic structures of transition metal compounds, in particular, ionic species, as evidenced in a study of fragmentation of a similar Cu(II) complex, [Cu(hfa)_2_TMEDA] [45]. For Ni, an “energy-adjusted” abinitio pseudopotential and (8s7p6d2f1g)/[6s5p3d2f1g] basis set were employed [55]. For all the other atoms, the D95(d) basis set was adopted, except for sodium, for which the 6–31G(d) set was employed [56]. All the computed optimized structures were characterized by no imaginary frequencies. The reported energy differences were calculated by including the zero-point-energy (ZPE) correction.

Bonding properties were theoretically investigated via the natural bond orbitals (NBOs) analysis [57]. In particular, the NBO charges on Ni, diketonate ligand L, and TMEDA reported in Table 3, the bond order values in Table 2 and Table 4, the quantitative NBO analyses in Tables S2–S4, as well as the natural bond orbitals depicted in Figures S3–S10, were obtained using the same combination of DFT functional/basis sets adopted in the geometry optimizations. The NBO analysis was performed by employing the NBO code (Version 3.1) available in Gaussian 09. Bond order values were calculated according to the Wiberg scheme [58]. 

Atomic charges reported in Tables S5–S8 were calculated through the Bader approach [59,60]. As in the case of the NBO analysis, the Bader charges were also computed using the same level of theory as adopted in the structural optimizations.

## 4. Conclusions

The chemical reactivity of three Ni(L)_2_TMEDA complexes (L = tfa, fod, thd) in decomposition processes was explored by adopting soft fragmentation procedures and using computational models. The combined experimental and theoretical analyses presented in this work, in addition to some ligand-dependent features, evidenced relevant common elements in the fragmentation pattern of the target compounds: (i) in cations formed in the first fragmentation, Ni-O and Ni-N interactions are stronger, and the positive charge on Ni is lower than in neutral complexes; (ii) the tendency of ligand electronic charge to progressively migrate towards Ni further increases in the subsequent fragmentation processes, leading to the formation of a tetracoordinated Ni environment involving C atoms belonging to partially fragmented TMEDA. From both the structural and electronic points of view, it is also interesting to note the formation of a C-C double bond in TMEDA rearrangements. As evidenced by NBO analysis and substantiated by the bond order trend, such a double bond is actually interacting with the Ni cation via an intramolecular cation-π interaction. In perspective, these analyses can be relevant for the understanding of the compounds’ reactivity under CVD conditions and shed light on the corresponding molecule-to-material conversion.

## Figures and Tables

**Table 1:** Main fragments, *m*/*z* ratios, and relative abundances (%) obtained in ESI-HRMS analyses of compounds **1**, **2**, and **3**. --- = not detected.

	1	2	3
Ionic Species	*m*/*z* (%)	*m*/*z* (%)	*m*/*z* (%)
[M+Na]^+^	---	787.1690 (25)	---
[M−TMEDA+Na]^+^	---	671.0379 (12)	---
[M−TMEDA+H]^+^	---	---	425.2190 (15)
[M−L]^+^	327.0830 (100)	469.1229 (100)	357.2041 (100)
[HL+H]^+^	---	---	185.1535 (18)
[TMEDA+H]^+^	117.1391 (70)	117.1389 (50)	117.1388 (55)

**Table 2:** Selected bond lengths (BL) (in Å) and corresponding bond orders (BO) from the optimized structures of neutral complexes M and fragments [M−L]^+^ for compounds **1**, **2**, and **3**. Different colors in the BO columns refer to different ligands (brown and violet = first and second diketonate ligand, respectively; gray = TMEDA). Atom labels as in Figure 3.

	1		2		3	
	M	[M-tfa]^+^	M	[M-fod]^+^	M	[M-thd]^+^
	BL BO	BL BO	BL BO	BL BO	BL BO	BL BO
Ni-O1	2.024 0.284	1.831 0.471	2.035 0.242	1.833 0.454	2.018 0.241	1.828 0.476
Ni-O4	2.024 0.284	-	2.035 0.242	-	2.018 0.241	-
Ni-O2	2.041 0.294	1.848 0.469	2.023 0.258	1.842 0.452	2.009 0.256	1.828 0.476
Ni-O3	2.041 0.294	-	2.023 0.258	-	2.010 0.256	-
Ni-N1	2.147 0.230	1.946 0.405	2.153 0.201	1.946 0.393	2.165 0.178	1.957 0.368
Ni-N2	2.147 0.230	1.946 0.399	2.153 0.201	1.947 0.387	2.165 0.178	1.957 0.368
O1-C5	1.260 1.362	1.276 1.285	1.260 1.363	1.276 1.279	1.267 1.352	1.284 1.267
O2-C7	1.257 1.427	1.274 1.318	1.258 1.418	1.276 1.316	1.263 1.376	1.284 1.267
O4-C6	1.260 1.362	-	1.260 1.363	-	1.267 1.352	-
O3-C8	1.257 1.427	-	1.258 1.418	-	1.263 1.376	-
C5-CH1	1.392 1.423	1.380 1.450	1.393 1.421	1.381 1.451	1.411 1.348	1.400 1.367
C7-CH1	1.424 1.264	1.413 1.300	1.425 1.260	1.413 1.288	1.414 1.325	1.400 1.367
C6-CH2	1.392 1.423	-	1.393 1.421	-	1.411 1.348	-
C8-CH2	1.424 1.264	-	1.425 1.260	-	1.414 1.325	-
CHT-CH2T	1.520 1.021	1.508 1.027	1.518 1.021	1.507 1.027	1.520 1.020	1.508 1.026

**Table 3:** NBO charges on Ni, diketonate ligand L, and on TMEDA computed for the optimized structures of neutral complexes M and fragments [M−L]^+^ for compounds **1**, **2**, and **3**. Different colors refer to different ligands (brown = diketonates, gray = TMEDA).

	1		2		3	
	M	[M-tfa]^+^	M	[M-fod]^+^	M	[M-thd]^+^
Ni	1.00	0.73	1.08	0.75	1.12	0.75
L	−0.65	−0.35	−0.68	−0.36	−0.69	−0.32
TMEDA	0.30	0.62	0.28	0.61	0.26	0.57

**Table 4:** Selected bond lengths (BL) (in Å) and corresponding bond orders (BO) from the optimized structures of fragments detected in ESI-MS^2^ spectra of [M−L]^+^ ions for compounds **1**, **2**, and **3**. Different colors in the BO columns refer to different ligands (brown = diketonate, gray = TMEDA). Atom labels as in Figure 3.

	1		2			3
	[M-tfa]^+^- NH(CH_3_)_2_	[M-tfa]^+^- CH_3_CH_2_N(CH_3_)_2_	[M-fod]^+^- NH(CH_3_)_2_	[M-fod]^+^- CH_3_CH_2_N(CH_3_)_2_	[M-fod]^+^- -(CH_3_)_2_C=CH_2_	[M-thd]^+^- NH(CH_3_)_2_
	BL BO	BL BO	BL BO	BL BO	BL BO	BL BO
Ni-O1	1.811 0.523	1.799 0.557	1.814 0.506	1.794 0.554	1.837 0.450	1.808 0.535
Ni-O2	1.830 0.511	1.819 0.541	1.816 0.518	1.815 0.543	1.842 0.453	1.803 0.546
Ni-N1	-	-	1.929 0.391	1.946 0.402	1.938 0.415	1.941 0.368
Ni-N2	1.932 0.393	1.943 0.408	-	-	1.937 0.414	-
Ni-CHT	1.972 0.248	1.987 0.264	1.976 0.239	1.986 0.264	-	1.974 0.243
Ni-CH2T	2.096 0.394	2.128 0.368	2.098 0.393	2.128 0.363	-	2.108 0.375
O1-C5	1.277 1.288	1.281 1.267	1.280 1.258	1.281 1.263	1.265 1.342	1.288 1.264
O2-C7	1.281 1.297	1.279 1.308	1.279 1.314	1.280 1.299	1.270 1.318	1.287 1.264
C5-CH1	1.384 1.444	1.381 1.463	1.380 1.468	1.381 1.458	1.400 1.345	1.399 1.381
C7-CH1	1.409 1.315	1.412 1.300	1.417 1.281	1.413 1.292	1.389 1.404	1.403 1.361
CHT-CH2T	1.379 1.566	1.373 1.588	1.380 1.567	1.373 1.590	1.508 1.027	1.378 1.579

## Data Availability

Data is contained within the article and Supplementary Material.

## Supplementary Materials

The following supporting information can be downloaded at: https://www.mdpi.com/article/10.3390/molecules29030642/s1. The supporting material file contains the graphical representations of minimum energy structures relevant for the fragmentation processes (Figures S1 and S2). Graphical representations of relevant Natural Bond Orbitals for diverse fragments (Figures S3–S10). Tables relative to relevant geometrical parameters and NBO analyses of fragments discussed in the main text (Tables S1–S4). Tables containing the atomic charges obtained via the Bader analysis (Tables S5–S8).
