# Supplementary material for: On the Fragmentation of Ni(II) β-Diketonate-Diamine Complexes as Molecular Precursors for NiO Films: A Theoretical and Experimental Investigation
Source: Molecules. 2024 Jan 30;29(3):642. doi: 10.3390/molecules29030642 (PMC10856068; doi:10.3390/molecules29030642)
Supplement: Supplementary file 1 [file molecules-29-00642-s001.zip › molecules-2809094-supplementary.pdf]

Supporting Material

On the Fragmentation of Ni(II) β-Diketonate-Diamine Complexes as Molecular Precursors for NiO Films: A Theoretical and Experimental Investigation

Cristiano Invernizzi [1], Gloria Tabacchi [1,*], Roberta Seraglia [2], Mattia Benedet [2,3], Marco Roverso [2,3], Chiara Maccato [2,3], Sara Bogialli [2,3], Davide Barreca [2,*] and Ettore Fois [1]

[1] Department of Science and High Technology, Insubria University and INSTM, 22100 Como, Italy; cinvernizzi@uninsubria.it (C.I.); ettore.fois@uninsubria.it (E.F.)
[2] CNR-ICMATE and INSTM, Department of Chemical Sciences, Padova University, 35131 Padova, Italy; roberta.seraglia@cnr.it (R.S.); mattia.benedet@phd.unipd.it (M.B.); marco.roverso@unipd.it (M.R.); chiara.maccato@unipd.it (C.M.); sara.bogialli@unipd.it (S.B.)
[3] Department of Chemical Sciences, Padova University and INSTM, 35131 Padova, Italy
* Correspondence: gloria.tabacchi@uninsubria.it (G.T.); davide.barreca@unipd.it (D.B.)

§ S1 Optimized structures of species detected in mass spectrometry experiments

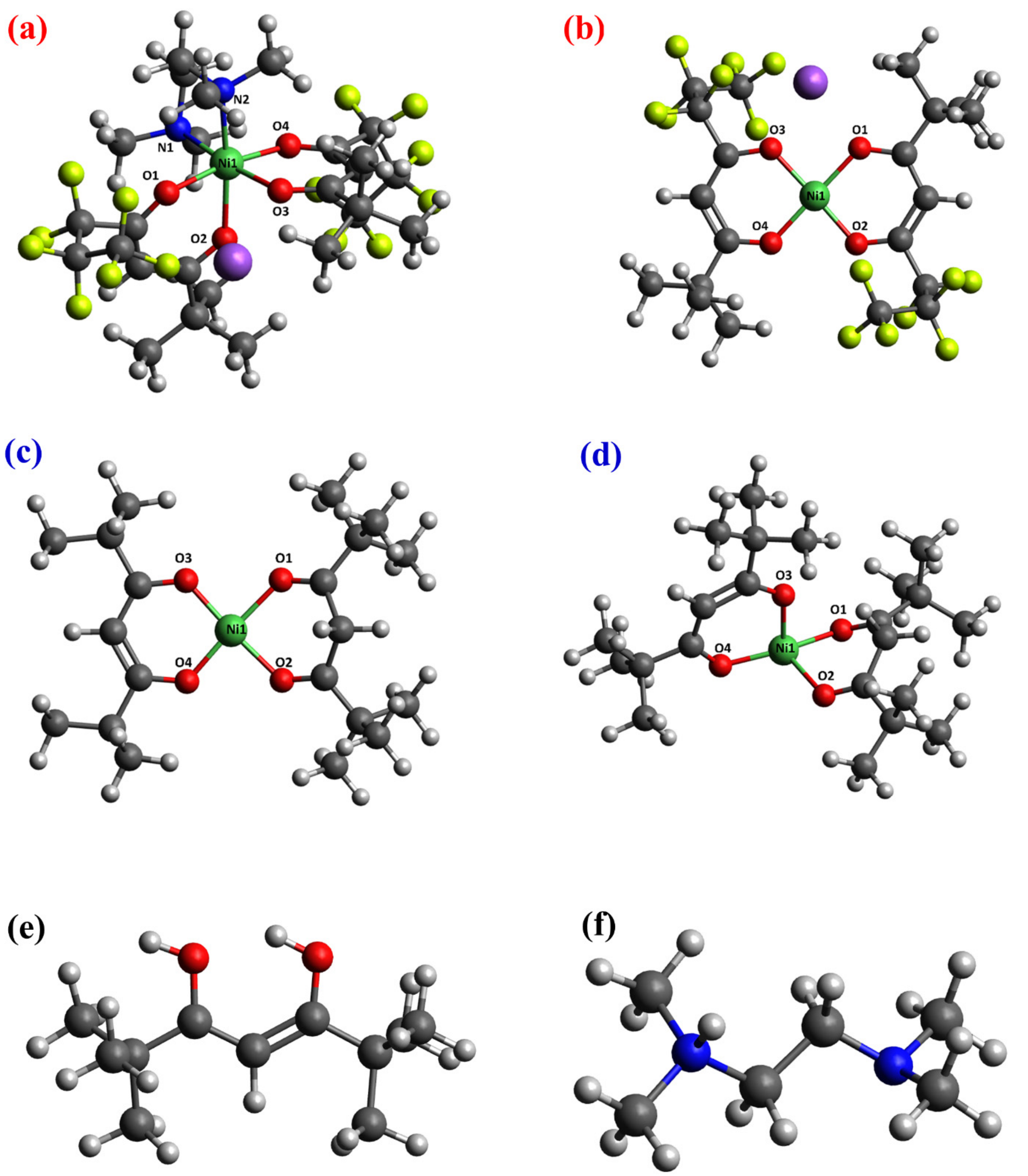

Figure S1. Graphical representation of the optimized structures of: (a) $[M+Na]^+ = [Ni(fod)_2TMEDA+Na]^+$, (triplet state); (b) $[M-TMEDA+Na]^+ = [Ni(fod)_2+Na]^+$, obtained by loss of TMEDA and addition of Na^+ from complex **2** ; (c) and (d) $[M-TMEDA+H]^+ = [Ni(thd)_2+H]^+$ pseudo-planar geometry (singlet state) and distorted tetrahedral geometry (triplet state), respectively. The second one is 0.32 kcal×mol^{-1} lower in energy with respect to (d); (e) $[HL+H]^+ = [thd+H]^+$ obtained by addition of H^+ to thd ligand; (f) $[TMEDA+H]^+$ obtained by addition of H^+ to neutral TMEDA. Color codes: purple = Na; green = Ni; yellow = F; blue = N; red = O; grey = C; white = H.

**Bond Lenghts (Å)**	**$[M+Na]^+$ (a)**	**$[M\text{-}TMEDA+Na]^+$ (b)**	**$[M\text{-}TMEDA+H]^+$ (c)**	**$[M\text{-}TMEDA+H]^+$ (d)**
Ni-O1	2.080	1.876	1.897	1.986
Ni-O2	2.112	1.822	1.897	1.981
Ni-O3	2.098	1.847	1.793	1.901
Ni-O4	1.989	1.837	1.793	1.842
Ni-N1	2.152	-	-	-
Ni-N2	2.126	-	-	-
Na-O1	2.502	2.389		
Na-O2	2.532	-		
Na-O3	2.215	2.240		
Na-F	2.441	2.542; 2.470		

Table S1. Ni-O and Ni-N bond distances of the species reported in Figure S1, i.e: (a) $[M+Na]^+ = [Ni(fod)_2TMEDA+Na]^+$, (triplet state); (b) $[M\text{-}TMEDA+Na]^+ = [Ni(fod)_2+Na]^+$, obtained from complex **2** by TMEDA loss and Na^+ addition; (c)-(d), $[M\text{-}TMEDA+H]^+ = [Ni(thd)_2+H]^+$ pseudo-planar geometry (singlet state) and distorted tetrahedral geometry (triplet state), respectively.

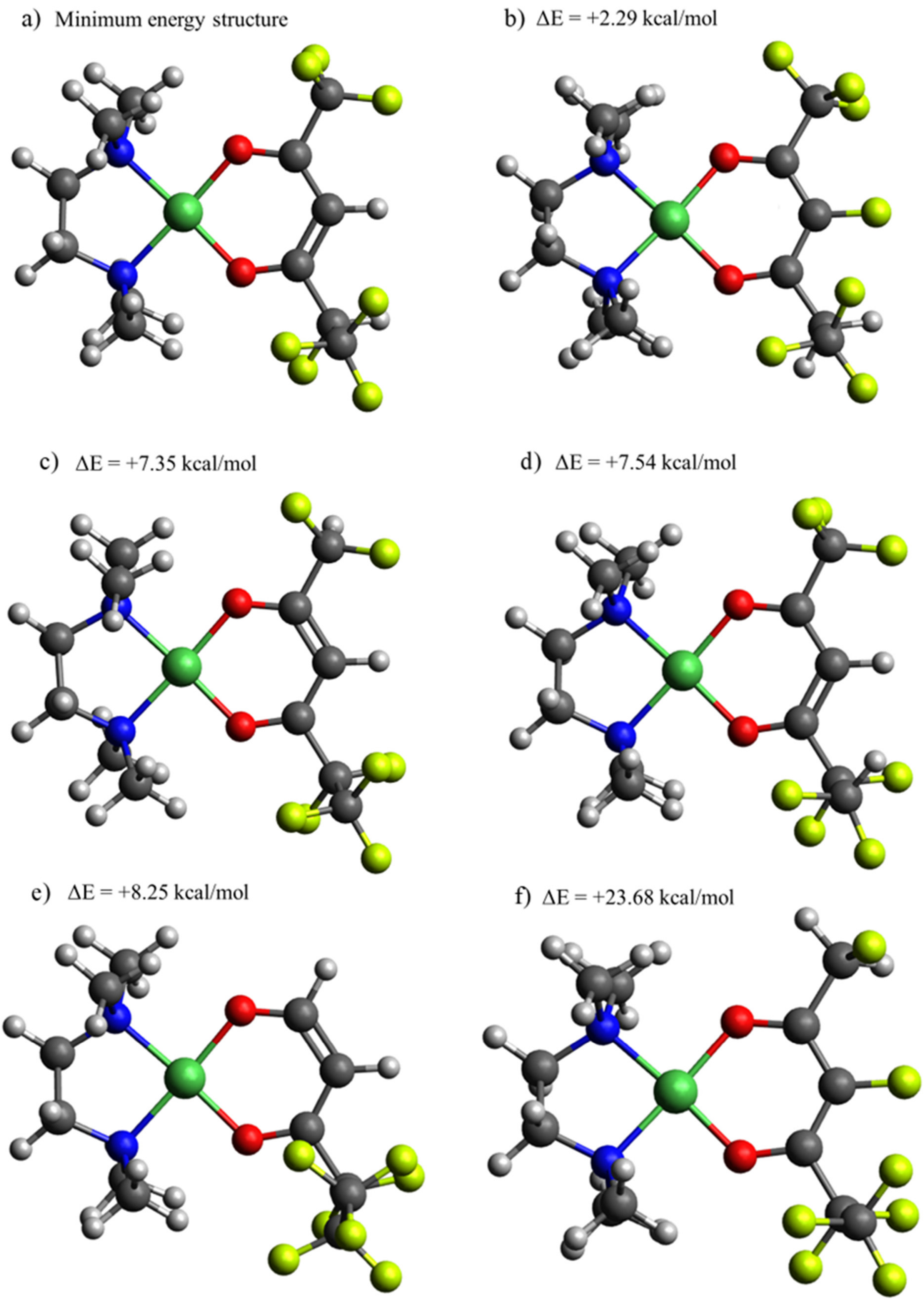

Figure S2. Graphical representation of six possible structures of fragment ion $[Ni(fod)TMEDA]^{+}$-$(CH_3)_2C{=}CH_2$, derived from an additional rearrangement of fod ligand and detected in MS^2 spectra of precursor **2**. Energy differences (relative energies) with respect to the minimum energy structure **(a)** are reported on the top of each model geometry. Color codes as in Figure S1.

§ S2. NBO analysis

Fragment	NBO type	Bond	Occupancy	% Ni	% X
**Ni(tfa)TMEDA⁺**					
	BD (σ)	*Ni-O2*	1.909	11	89
	BD (σ)	*Ni-O1*	1.904	11	89
	BD (σ)	*Ni-N1*	1.866	12	88
	BD (σ)	*Ni-N2*	1.868	12	88
	BD* (σ)	*Ni-O2*	0.100	89	11
	BD* (σ)	*Ni-O1*	0.102	89	11
	BD* (σ)	*Ni-N1*	0.128	88	12
	BD* (σ)	*Ni-N2*	0.128	88	12
**Ni(fod)TMEDA⁺**					
	BD (σ)	*Ni-O2*	1.903	11	89
	BD (σ)	*Ni-O1*	1.897	11	89
	BD (σ)	*Ni-N1*	1.861	11	89
	BD (σ)	*Ni-N2*	1.863	11	89
	BD* (σ)	*Ni-O2*	0.111	89	11
	BD* (σ)	*Ni-O1*	0.112	89	11
	BD* (σ)	*Ni-N1*	0.132	89	11
	BD* (σ)	*Ni-N2*	0.133	89	11
**Ni(thd)TMEDA⁺**					
	BD (σ)	*Ni-O2*	1.896	11	89
	BD (σ)	*Ni-O1*	1.896	11	89
	BD (σ)	*Ni-N1*	1.866	10	90
	BD (σ)	*Ni-N2*	1.866	10	90
	BD* (σ)	*Ni-O2*	0.110	89	11
	BD* (σ)	*Ni-O1*	0.110	89	11
	BD* (σ)	*Ni-N1*	0.137	90	10
	BD* (σ)	*Ni-N2*	0.137	90	10

Table S2. Natural bond orbital (NBO) analysis for $[Ni(L)TMEDA]^+$, where L = tfa, fod, thd, reporting: NBO type, atoms on which NBOs are localized, NBO occupancy, and localization (%) of NBOs on the involved atoms. BD = bonding character; BD* = anti-bonding character; X = O or N. Atoms labels as in Figure 3.

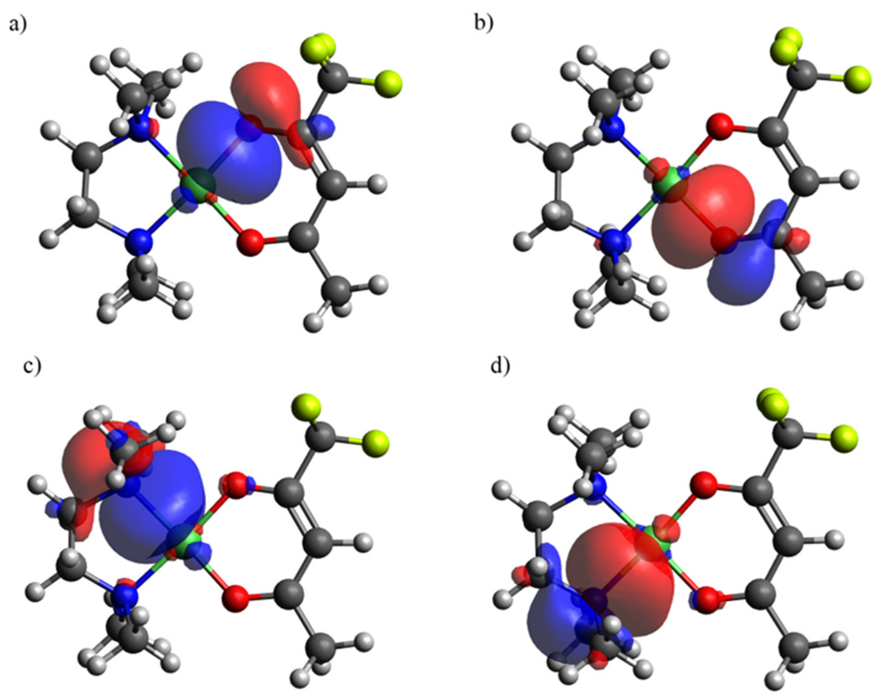

Figure S3. Graphical representation of natural bond orbitals of [Ni(tfa)TMEDA]$^+$ localized on the following bonds: a) Ni-O1 (BD); b) Ni-O2 (BD); c) Ni-N1 (BD); d) Ni-N2 (BD) (see also Table S2). Atom color codes: green = Ni; yellow = F; blue = N; red = O; grey = C; white = H.

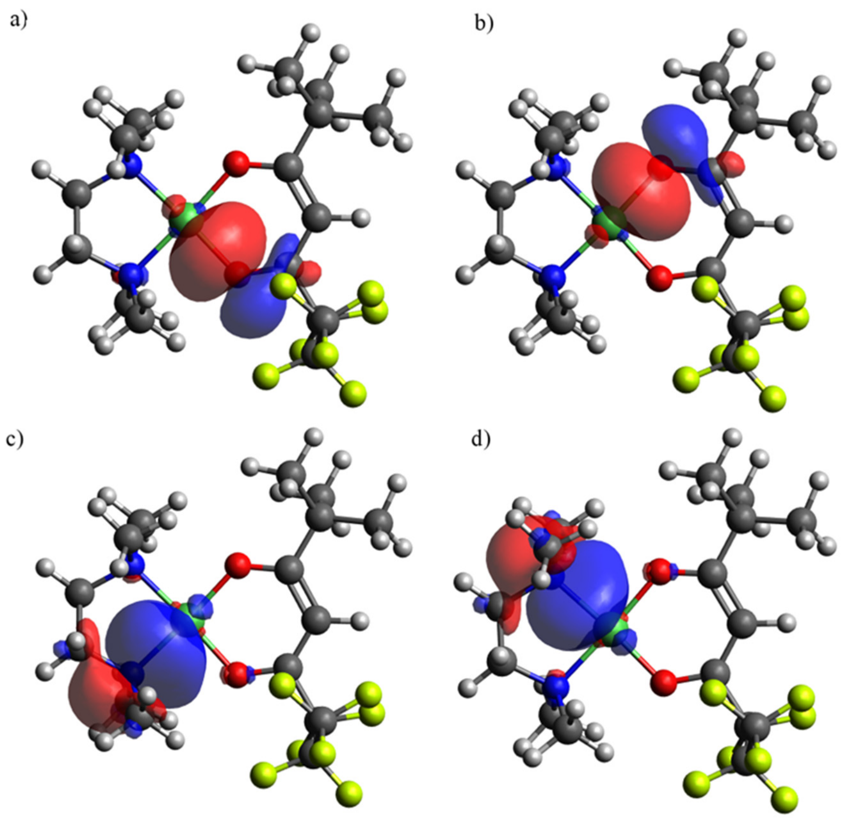

Figure S4. Graphical representation of natural bond orbitals of [Ni(fod)TMEDA]$^+$ localized on the following bonds: a) Ni-O1 (BD); b) Ni-O2 (BD); c) Ni-N1 (BD); d) Ni-N2 (BD) (see also Table S2). Atom color codes as in Figure S3.

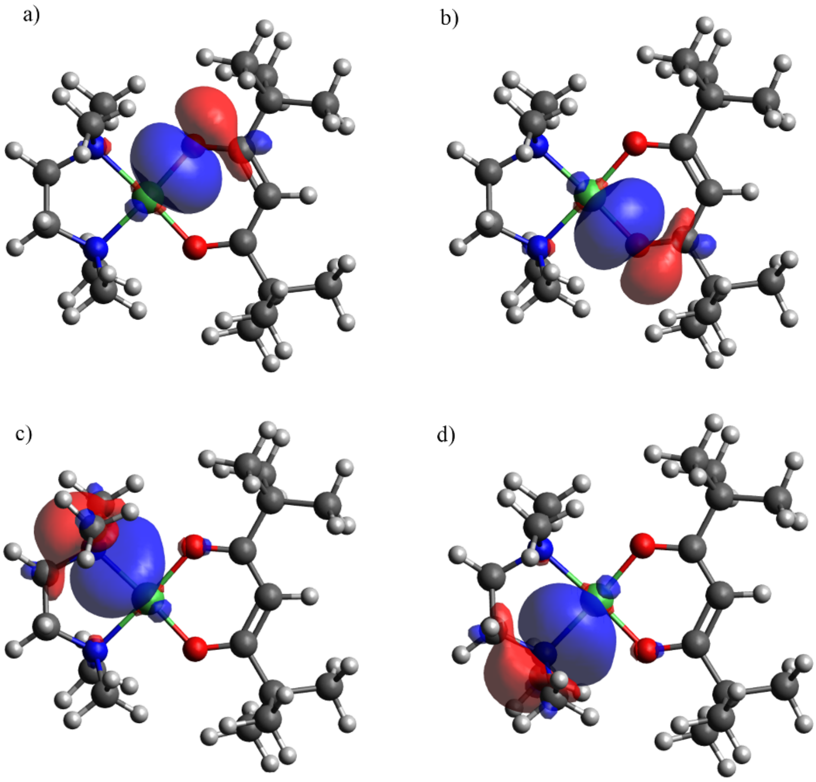

Figure S5. Graphical representation of natural bond orbitals of $[Ni(thd)TMEDA]^+$ localized on the following bonds: a) Ni-O1 (BD); b) Ni-O2 (BD); c) Ni-N1 (BD); d) Ni-N2 (BD) (see also Table S2). Atom color codes: green = Ni; blue = N; red = O; grey = C; white = H.

Fragment	NBO type	Bond	Occupancy	% Y	% X
Ni(tfa)TMEDA$^+$ -NH(CH_3)$_2$					
	BD (π)	*CHT-CH2T*	1.985	52	48
	BD (π)	*CHT-CH2T*	1.663	49	51
	BD (σ)	*Ni-N1*	1.878	26	74
	BD* (π)	*CHT-CH2T*	0.011	48	52
	BD* (π)	*CHT-CH2T*	0.187	51	49
	BD* (σ)	*Ni-N1*	0.527	74	26
Ni(fod)TMEDA$^+$ -NH(CH_3)$_2$					
	BD (π)	*CHT-CH2T*	1.981	52	48
	LP (π)	*CH2T*	0.959	-	-
	BD (σ)	*CHT-N1*	1.749	22	78
	BD* (π)	*CHT-CH2T*	0.010	48	52
	BD* (σ)	*CHT-N1*	0.676	78	22
Ni(thd)TMEDA$^+$ -NH(CH_3)$_2$					
	BD (π)	*CHT-CH2T*	1.985	52	48
	BD (π)	*CHT-CH2T*	1.691	49	51
	BD (σ)	*Ni-N1*	1.871	24	76
	BD* (π)	*CHT-CH2T*	0.011	48	52
	BD* (π)	*CHT-CH2T*	0.198	51	49
	BD* (σ)	*Ni-N1*	0.548	76	24

Table S3. NBO analysis for [Ni(L)TMEDA]$^+$-NH(CH_3)$_2$, where L = tfa, fod, thd. The table reports: NBO type, atoms on which NBOs are localized, NBOs occupancy and the percentage localization of NBOs on the involved atoms. BD = bonding; BD* = anti-bonding; LP = lone-pair; Y = Ni or CHT; X = N1 or CH2T. Atoms labels as in Figure 4.

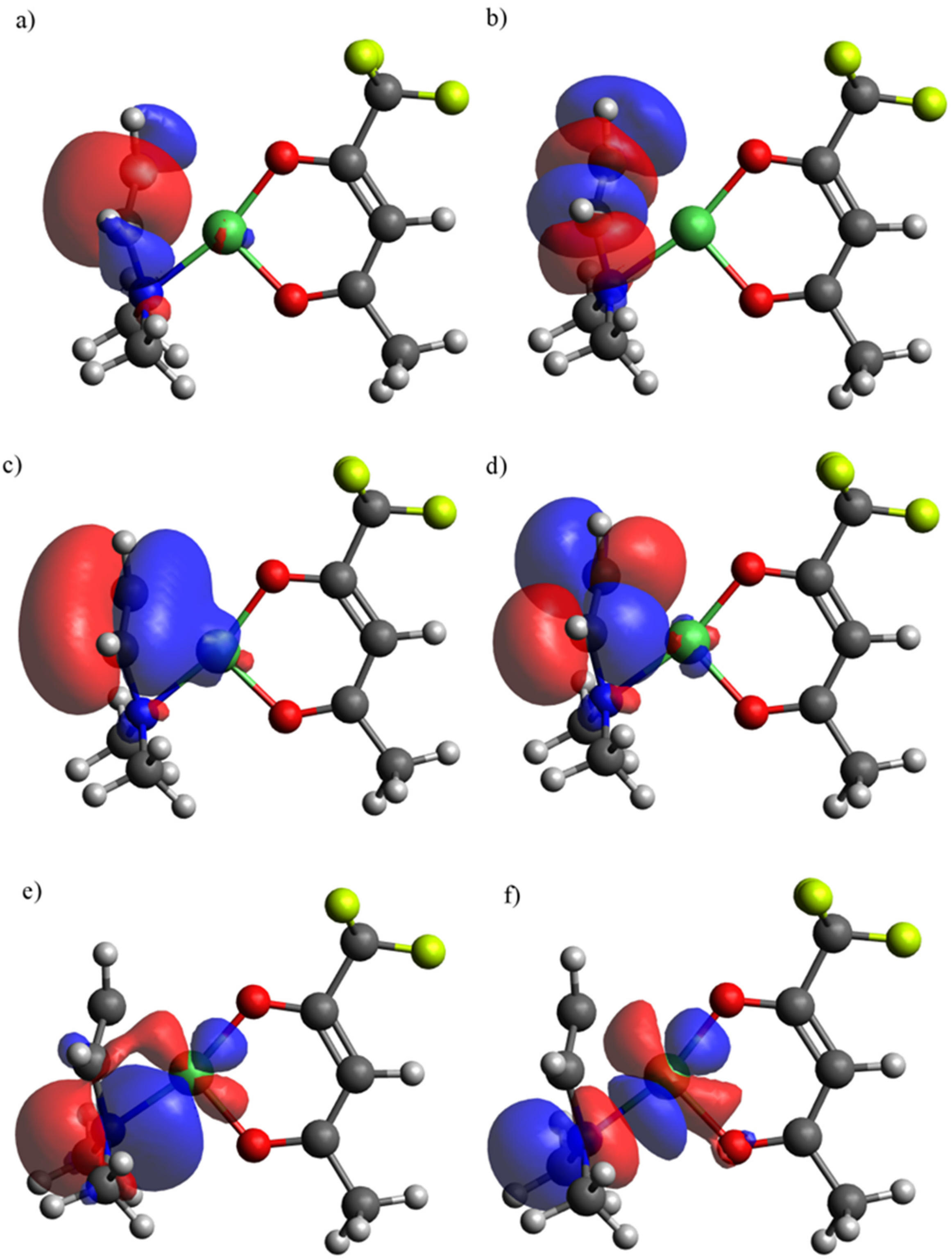

Figure S6. Graphical representation of natural bond orbitals of $[Ni(tfa)TMEDA]^{+}$-$NH(CH_3)_2$ localized on the following bonds: a) CHT-CH2T (BD); b) CHT-CH2T (BD*); c) CHT-CH2T (BD); d) CHT-CH2T (BD*); e) Ni-N1 (BD); f) Ni-N1 (BD*) (see also Table S3). Atom color codes as in Figure S3.

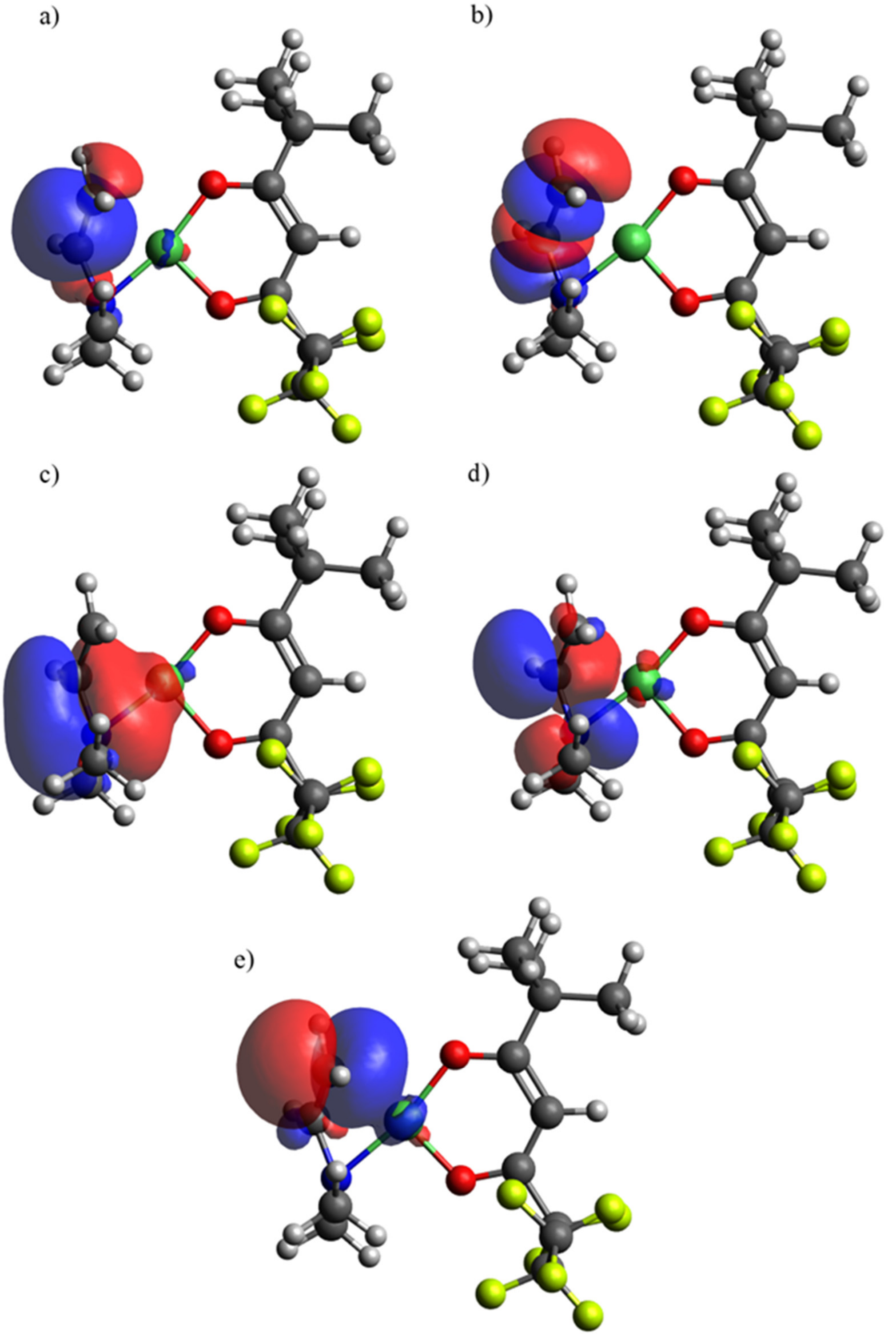

Figure S7. Graphical representation of natural bond orbitals of [Ni(fod)TMEDA]$^+$-NH(CH_3)$_2$ localized on the following bonds: a) CHT-CH2T (BD); b) CHT-CH2T (BD*); c) Ni-N1 (BD); d) Ni-N1 (BD*); e) CH2T (LP) (see also Table S3). Atom color codes as in Figure S3.

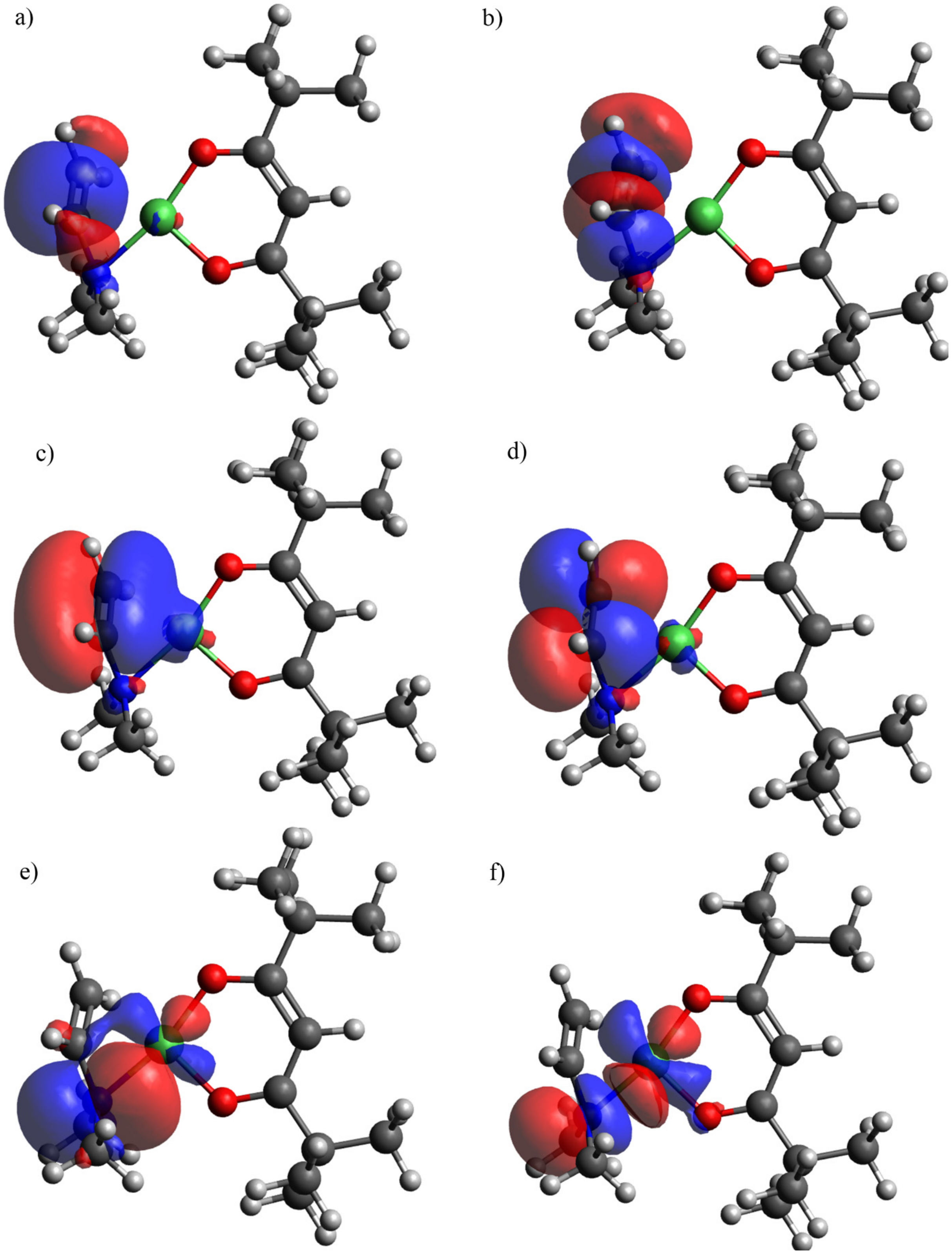

Figure S8. Graphical representation of natural bond orbitals of [Ni(thd)TMEDA]$^{+}$-NH(CH_3)$_2$ localized on the following bonds: a) CHT-CH2T (BD); b) CHT-CH2T (BD*); c) CHT-CH2T (BD); d) CHT-CH2T (BD*); e) Ni-N1 (BD); f) Ni-N1 (BD*) (see also Table S3). Atom color codes as in Figure S5.

Fragment	NBO type	Bond	Occupancy	% Y	% X
**Ni(tfa)TMEDA⁺ -$CH_3CH_2N(CH_3)_2$**					
	BD (π)	*CHT-CH2T*	1.987	52	48
	LP (π)	*CHT*	0.916	-	-
	LP (π)	*CH2T*	0.914	-	-
	BD (σ)	*Ni-N1*	1.836	14	86
	BD* (π)	*CHT-CH2T*	0.010	48	52
	BD* (σ)	*Ni-N1*	0.197	86	14
**Ni(fod)TMEDA⁺ - $CH_3CH_2N(CH_3)_2$**					
	BD (π)	*CHT-CH2T*	1.987	52	48
	LP (π)	*CHT*	0.920	-	-
	LP (π)	*CH2T*	0.914	-	-
	BD (σ)	*Ni-N1*	1.838	14	86
	BD* (π)	*CHT-CH2T*	0.010	48	52
	BD* (σ)	*Ni-N1*	0.197	86	14

Table S4. NBO analysis for $[Ni(L)TMEDA]^+$-$CH_3CH_2N(CH_3)_2$, where L = tfa and fod. The table shows the NBO type, the atoms on which NBOs are localized, NBO occupancy and the percentage localization of NBOs on the involved atoms. BD = bonding; BD* = anti-bonding; LP = lone-pair; Y = Ni or CHT; X = N1 or CH2T. Atoms labels as in Figure 4.

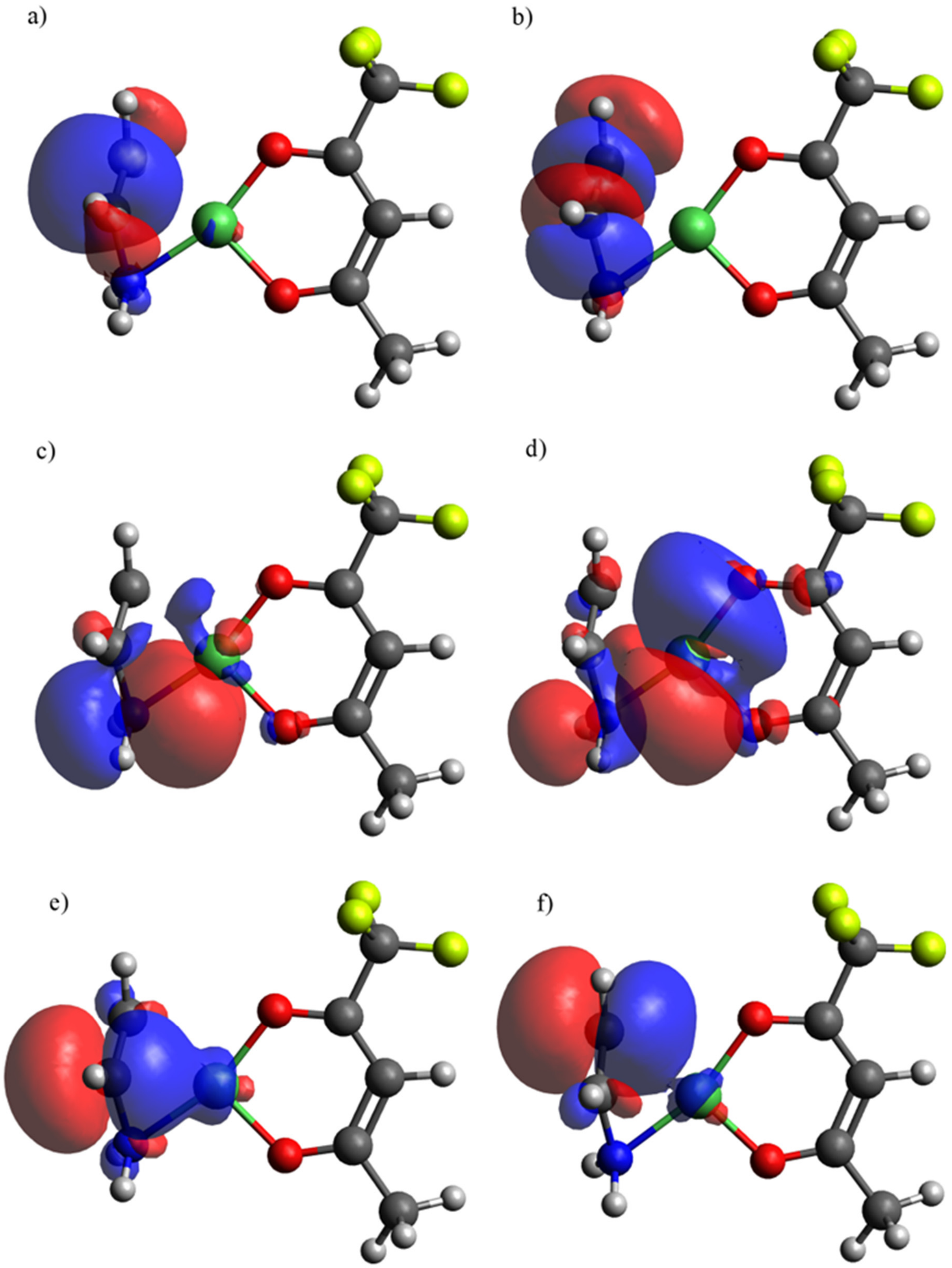

Figure S9. Graphical representation of natural bond orbitals of [Ni(tfa)TMEDA]$^+$-$CH_3CH_2N(CH_3)_2$ localized on the following bonds: a) CHT-CH2T (BD); b) CHT-CH2T (BD*); c) Ni-N1 (BD); d) Ni-N1 (BD*); e) CHT (LP); f) CH2T (LP) (see also Table S4). Atom color codes as in Figure S3.

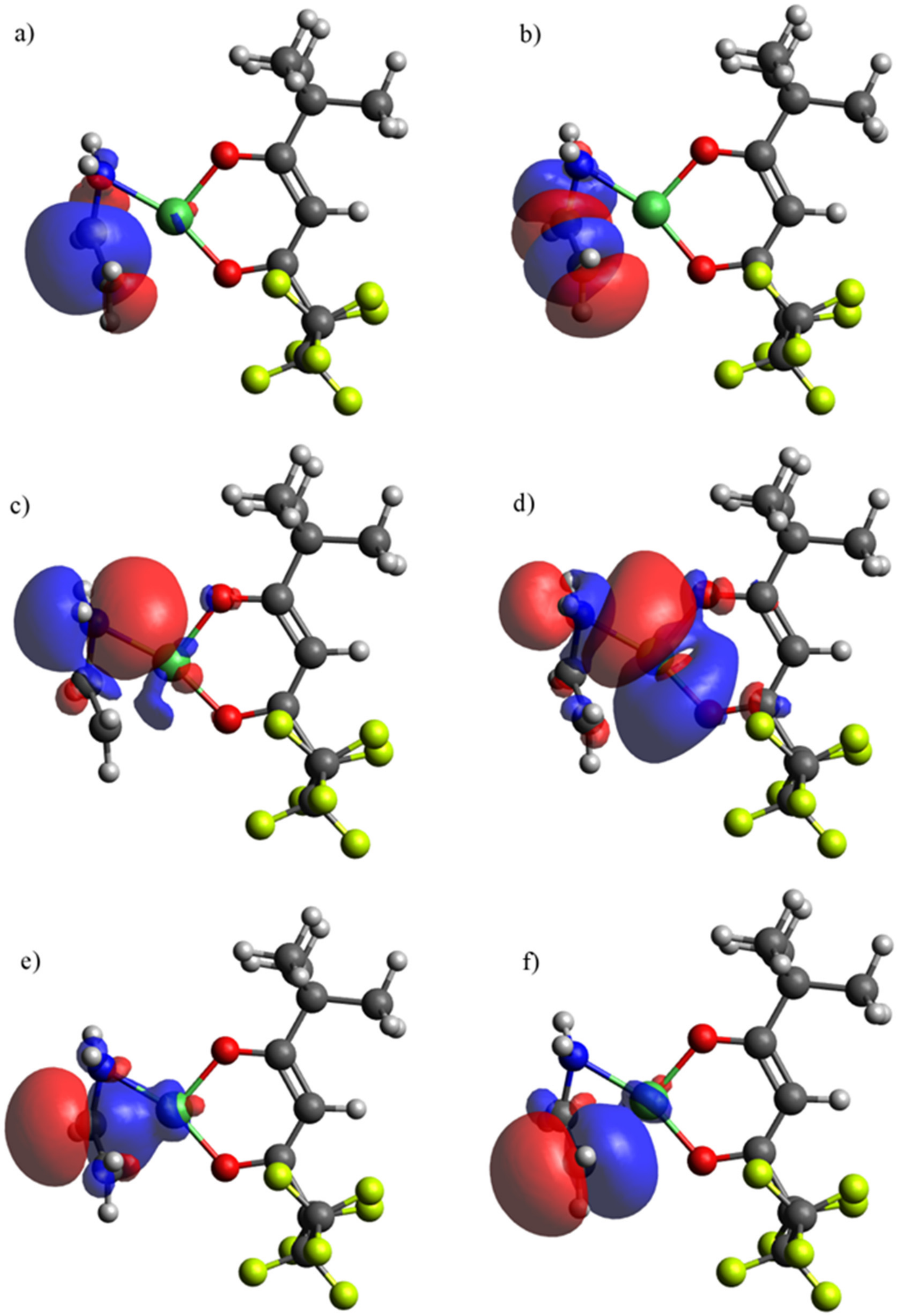

Figure S10. Graphical representation of natural bond orbitals of [Ni(fod)TMEDA]$^+$-$CH_3CH_2N(CH_3)_2$ localized on the following bonds: a) CHT-CH2T (BD); b) CHT-CH2T (BD*); c) Ni-N1 (BD); d) Ni-N1 (BD*); e) CHT (LP); f) CH2T (LP) (see also Table S4). Atom color codes as in Figure S3.

**Atom**	**Ni(tfa)$_2$TMEDA**	**Ni(fod)$_2$TMEDA**	**Ni(thd)$_2$TMEDA**
Ni	+1.33	+1.33	+1.33
O1	-1.19	-1.18	-1.21
O2	-1.20	-1.19	-1.21
O3	-1.20	-1.20	-1.19
O4	-1.19	-1.18	-1.21
N1	-1.03	-1.03	-1.04
N2	-1.05	-1.05	-1.04
C5	+0.81	+0.81	+0.78
C6	+0.82	+0.77	+0.77
C7	+0.86	+0.84	+0.78
C8	+0.86	+0.85	+0.78
CH1	-0.01	-0.03	-0.07
CH2	-0.03	0.00	-0.05
CHT	+0.27	+0.25	+0.28
CH2T	+0.31	+0.28	+0.28

Table S5. Bader charges for [Ni(L)$_2$TMEDA], where L = tfa, fod, thd. Atom labels as in Figure 3.

**Atom**	**[Ni(tfa)TMEDA]$^+$**	**[Ni(fod)TMEDA]$^+$**	**[Ni(thd)TMEDA]$^+$**
Ni	+0.99	+1.00	+1.00
O1	-1.14	-1.16	-1.16
O2	-1.14	-1.17	-1.17
N1	-1.03	-1.01	-1.01
N2	-1.01	-1.03	-1.03
C5	+0.71	+0.67	+0.67
C7	+0.72	+0.65	+0.65
CH1	+0.10	+0.08	+0.08
CHT	+0.28	+0.27	+0.27
CH$_2$T	+0.28	+0.27	+0.27

Table S6. Bader charges for [Ni(L)TMEDA]$^+$, where L = tfa, fod, thd. Atom labels as in Figure 3.

Atom	$[Ni(tfa)TMEDA]^+$ - $NH(CH_3)_2$	$[Ni(fod)TMEDA]^+$ - $NH(CH_3)_2$	$[Ni(thd)TMEDA]^+$ - $NH(CH_3)_2$
Ni	+0.95	+0.95	+0.95
O1	-1.12	-1.13	-1.13
O2	-1.17	-1.12	-1.12
N	-1.09	-1.10	-1.10
C5	+0.70	+0.67	+0.67
C7	+0.72	+0.71	+0.71
CH1	+0.09	+0.09	+0.09
CHT	+0.22	+0.21	+0.21
CH_2T	-0.05	-0.08	-0.08

Table S7. Bader charges for $[Ni(L)TMEDA]^+$ -$NH(CH_3)_2$, where L = tfa, fod, thd. Atom labels as in Figure 4. N indicates the nitrogen atom bound to Ni.

Atom	$[Ni(tfa)TMEDA]^+$ - $CH_3CH_2N(CH_3)_2$	$[Ni(fod)TMEDA]^+$ - $CH_3CH_2N(CH_3)_2$	$[Ni(fod)TMEDA]^+$ - $(CH_3)_2C{=}CH_2$
Ni	+0.97	+0.97	+1.00
O1	-1.11	-1.09	-1.12
O2	-1.12	-1.13	-1.14
N	-1.15	-1.14	-1.01
C5	+0.68	+0.68	+0.76
C7	+0.72	+0.69	+0.77
CH1	+0.09	+0.08	+0.07
CHT	+0.21	+0.19	+0.27
CH_2T	-0.06	-0.05	+0.24

Table S8. Bader charges for $[Ni(L)TMEDA]^+$ - $CH_3CH_2N(CH_3)_2$, where L = tfa, fod, and for $[Ni(fod)TMEDA]^+$-$(CH_3)_2C{=}CH_2$. Atom labels as in Figure 4. In each case, N indicates the nitrogen atom(s) bound to Ni (for $[Ni(fod)TMEDA]^+$-$(CH_3)_2C{=}CH_2$, both N atoms are ligated to Ni and bear identical Bader charges).
